# Aortic Elasticity and Cardiac Electrophysiological Balance in Opioid Users Receiving Buprenorphine/Naloxone Maintenance Therapy

**DOI:** 10.3390/biomedicines14030700

**Published:** 2026-03-17

**Authors:** Azmi Eyiol, Hatice Eyiol, Ahmet Yılmaz, Emine Hande Kilicaslan Sahin, Yakup Alsancak

**Affiliations:** 1 Department of Cardiology, Beyhekim Training and Research Hospital, 42130 Konya, Turkey; 2 Department of Anesthesiology and Reanimation, Beyhekim Training and Research Hospital, 42100 Konya, Turkey; hatice.eyiol@saglik.gov.tr; 3 Department of Cardiology, Faculty of Medicine, Karamanoğlu Mehmetbey University, 70100 Karaman, Turkey; drahmetyilmaz@kmu.edu.tr; 4 Department of Psychiatry, Beyhekim Training and Research Hospital, 42100 Konya, Turkey; emine.kilicaslan@saglik.gov.tr; 5 Department of Cardiology, Faculty of Medicine, Necmettin Erbakan University, 42090 Konya, Turkey; yakupalsancak@erbakan.edu.tr

**Keywords:** opioid dependence, aortic elasticity, aortic stiffness, iCEB, buprenorphine/naloxone, cardiovascular risk

## Abstract

**Background**: Opioid dependence treated with buprenorphine/naloxone is associated with increased cardiovascular risk; however, data regarding aortic elasticity and cardiac electrophysiological balance during long-term maintenance therapy remain limited. This study investigated aortic stiffness and distensibility in individuals receiving buprenorphine/naloxone (Suboxone), and examined their associations with echocardiographic and electrocardiographic parameters, including the index of cardiac electrophysiological balance (iCEB and iCEBc). **Methods**: A retrospective cohort analysis was conducted, including 130 intravenous opioid users receiving Suboxone and 150 age- and sex-matched healthy controls. All participants underwent clinical evaluation, transthoracic echocardiography, resting 12-lead electrocardiography, and 24-h ambulatory blood pressure monitoring. **Results**: Compared to controls, opioid users demonstrated significantly higher aortic distensibility (median 0.019 vs. 0.015, *p* < 0.001) and lower aortic stiffness (median 52.31 vs. 64.66, *p* < 0.001). Patients receiving Suboxone for more than 18 months exhibited higher diastolic blood pressure (*p* = 0.044), mean arterial pressure (*p* = 0.046), and pulmonary artery pressure (*p* = 0.022). Aortic elasticity indices showed strong correlations with blood pressure and echocardiographic parameters. In the overall cohort, Suboxone use duration was not significantly correlated with aortic stiffness or distensibility parameters, while a weak negative correlation was observed with ferritin levels (r = −0.231, *p* = 0.008). In subgroup analysis of long-term users (>18 months), a moderate positive correlation was observed between therapy duration and iCEB values (r = 0.367, *p* = 0.001). **Conclusions**: Chronic buprenorphine/naloxone therapy appears to be associated with changes in aortic elasticity, blood pressure, and mild electrophysiological alterations. These results support the use of non-invasive vascular and electrocardiographic evaluations for cardiovascular risk monitoring and stratification among patients receiving opioid maintenance therapy.

## 1. Introduction

Opioid use disorder (OUD) has become a public health crisis in the United States, contributing significantly to morbidity, mortality, lost productivity, and criminal justice system costs [1,2]. In 2018, at least 2 million individuals in the U.S. were diagnosed with a substance use disorder related to prescription opioid analgesics [3]. Patients receiving care within the Veterans Health Administration have been disproportionately affected, with a sevenfold higher prevalence of OUD treatment compared to those covered by commercial health plans [4]. Opioid overdose deaths reached a record high of 50,042 in 2019, and this number likely increased in 2020 due to the COVID-19 pandemic, which exacerbated substance use, heightened stress, intensified social isolation, and disrupted access to opioid treatment [5]. Individuals with opioid use disorder also represent a major global health burden associated with both direct and indirect complications [6]. Opioid use, including buprenorphine/naloxone maintenance therapy, has been linked to alterations in autonomic function, endothelial dysfunction, and an increased risk of cardiovascular events [7]. Chronic substance use can contribute to arterial stiffness, increased aortic workload, and adverse vascular remodeling, necessitating comprehensive cardiovascular assessment in these patients [8].

Buprenorphine is considered the first-line treatment for opioid use disorder in the United States and Canada [9]. Its use has been associated with a 3.3-fold reduction in overdose mortality and is regarded as a key component in addressing the rising rates of opioid toxicity. Although the initial formulation was approved in the U.S. in 2002 and in Canada in 2007, the combination product buprenorphine–naloxone remains underutilized. In our study, all patients received buprenorphine/naloxone.

Aortic stiffness and aortic distensibility are key markers of vascular health, reflecting arterial elasticity and the ability to accommodate pulsatile blood flow [10,11]. Increased aortic stiffness is an independent predictor of adverse cardiovascular outcomes, including hypertension, heart failure, and atherosclerosis [8]. Echocardiography, electrocardiography (ECG), and ambulatory blood pressure monitoring (ABPM) are widely used non-invasive methods for assessing cardiovascular function in various clinical populations, including those with opioid use disorders [12]. However, data on aortic stiffness and distensibility in individuals using maintenance therapy are limited.

Recent advancements in electrocardiographic analysis have introduced the Index of Cardiac Electrophysiological Balance (iCEB), calculated as the ratio of the QT interval to the QRS duration (QT/QRS), as a promising marker for ventricular repolarization balance and arrhythmic risk. Its heart rate-corrected form, iCEBc (QTc/QRS), offers improved comparability across variable heart rates [13]. These indices are thought to reflect the myocardial electrical stability and have shown potential in predicting malignant ventricular arrhythmias in various cardiac conditions. For instance, extrinsic alterations in cardiac repolarization due to ischemia, inflammation, or autonomic imbalance may influence iCEB and iCEBc values, thus providing indirect information about structural and functional myocardial integrity. However, their role in populations with substance use disorders, especially in relation to vascular stiffness and elasticity, remains poorly understood.

Given the known effects of chronic opioid use and maintenance therapy on autonomic tone, endothelial function, and vascular remodeling, it is plausible that electrophysiological markers such as iCEB and iCEBc may also reflect these alterations. Arterial stiffness and reduced distensibility may impact myocardial afterload, wall stress, and oxygen demand, all of which could modulate repolarization dynamics captured on surface ECGs. In a recent study, impaired aortic elasticity was associated with prolonged repolarization parameters, suggesting a mechanistic link between vascular and electrical remodeling in chronic cardiovascular stress conditions [14]. Therefore, evaluating the relationship between aortic stiffness/distensibility and iCEB/iCEBc may offer novel insights into the cardiovascular risk stratification of opioid-dependent individuals receiving maintenance therapy, with potential implications for early cardiovascular risk identification and stratification in opioid-dependent populations.

This study aims to evaluate aortic stiffness and distensibility, as well as cardiac electrophysiological balance (iCEB and iCEBc), in opioid-dependent individuals receiving buprenorphine/naloxone maintenance therapy.

## 2. Material and Methods

### 2.1. Compliance with Ethical Standards

The study was reviewed and approved by the institutional research ethics board in accordance with the principles of the Declaration of Helsinki. We received ethics committee approval from Konya Necmettin Erbakan University Ethics committee. Ethics committee approval for the study was received with the decision of the university committee meeting dated 7 February 2025 and numbered 2025/5515.

### 2.2. Study Design

This study was designed as a retrospective cohort study, including a total of 130 patients diagnosed with opioid users between January 2023 and January 2025. Data were obtained from hospital electronic health records, including laboratory results, ECG reports, echocardiographic findings, and treatment records. Patient charts were retrospectively reviewed to collect data on hemoglobin levels, red cell distribution width (RDW), albumin levels, ECG findings, echocardiographic parameters, treatment strategies, and duration of opioid use. iCEB was analyzed by the ratio of QT/QRS. iCEBc was analyzed by the ratio of QTc/QRS. QT and QRS intervals were manually measured from standard 12-lead resting ECGs recorded at a paper speed of 25 mm/s and amplitude of 10 mm/mV. Lead II was used as the primary reference for all interval measurements due to its clarity and reproducibility. All measurements were performed using digital calipers on scanned high-resolution ECG images. For each patient, three consecutive sinus beats were analyzed, and the average values were calculated. QT intervals were measured from the onset of the QRS complex to the end of the T wave, defined as the return to the isoelectric line. Two independent cardiologists performed the measurements, and discrepancies were resolved by consensus. No automated software was utilized in the measurement process. As this study was retrospective in design, no prior sample size calculation was performed. The sample size was based on the available population meeting the inclusion criteria over the two-year period.

### 2.3. Patient Evaluation and Follow-Up

This study included patients receiving maintenance therapy for opioid dependence who underwent comprehensive cardiovascular assessments. Inclusion criteria were as follows: patients aged 18–65 years with a history of opioid use and stable maintenance treatment for at least three months. Exclusion criteria included known cardiovascular disease (such as coronary artery disease, heart failure, or valvular heart disease), uncontrolled hypertension, diabetes mellitus, chronic kidney or liver disease, active infections, autoimmune disorders, malignancies, or the use of medications affecting vascular function. Patients with inadequate echocardiographic image quality or incomplete follow-up data were also excluded.

Although the eligibility criteria allowed inclusion of patients aged between 18 and 65 years, the actual age distribution of the study cohort was considerably narrower. Most individuals receiving opioid maintenance therapy at our center were young adults, which resulted in a relatively homogeneous age distribution in the analyzed population (median age 28 years, IQR 25–31). To minimize the potential confounding effect of age on vascular parameters, the control group was matched to the opioid group by age (±2 years) and sex.

The control group consisted of 150 age- and sex-matched individuals who underwent routine health evaluations at our hospital during the same time period as the patient cohort. Matching was performed based on age (±2 years) and sex distribution. Control subjects were screened using standardized medical history forms and interviews to confirm the absence of opioid use, tobacco smoking, alcohol consumption, or other psychoactive substance use. Individuals with a history of cardiovascular disease, diabetes, hypertension, chronic kidney or liver disease, autoimmune or inflammatory disorders, or use of medications affecting vascular function were excluded. The same exclusion criteria applied to the opioid group were also applied to controls to minimize potential confounding factors.

The 18-month threshold corresponded approximately to the median treatment duration within the study cohort and was selected to create two comparable subgroups for exploratory analysis.

Smoking status was assessed through structured clinical interviews and review of medical records documented during routine addiction treatment follow-up visits. Individuals with documented active tobacco use were excluded from both the opioid and control groups to reduce the well-known confounding effect of smoking on arterial stiffness parameters.

For exploratory subgroup analyses, patients were categorized according to the duration of Suboxone therapy (≤18 months vs. >18 months). The 18-month threshold corresponded approximately to the median duration of treatment within our cohort and was selected to create two comparable groups for analysis.

Information regarding concurrent illicit substance use was obtained from psychiatric evaluations, clinical interviews, and routine toxicology screening results recorded in the hospital database. Patients with documented use of other illicit substances during the follow-up period were excluded from the analysis.

Eligible participants underwent a detailed medical history assessment, including opioid use patterns, comorbidities, and medication history. A physical examination was performed, followed by resting ECG, transthoracic echocardiography (TTE), and 24-h ABPM to evaluate cardiac function, vascular parameters, and autonomic regulation. Twenty-four-hour ambulatory blood pressure monitoring was performed using a validated oscillometric device (Mobil-O-Graph NG, I.E.M. GmbH, Stolberg, Germany), with measurements obtained at 20-min intervals during daytime and 30-min intervals during nighttime. Blood samples were collected for laboratory analysis, including hemoglobin, RDW, albumin levels, and routine biochemical markers.

ECG measurements were performed independently by two experienced cardiologists. If discrepancies greater than 5 ms for ECG intervals or greater than 1 mm for echocardiographic diameter measurements were detected, the images were re-evaluated jointly and a consensus value was determined after repeat inspection of the original ECG tracing or echocardiographic image.

Aortic stiffness and aortic distensibility were assessed using echocardiographic parameters and ABPM data. Hemodynamic parameters such as systolic and diastolic blood pressure variability were recorded. Patients were categorized based on the presence or absence of increased aortic stiffness, and comparisons were made between groups. Follow-up assessments ensured data consistency and allowed for the exclusion of confounding factors.

Aortic stiffness, aortic distensibility, and aortic strain were calculated using the following formulas:

- Aortic stiffness: ln(SBP/DBP)/[(Systolic aortic diameter − Diastolic aortic diameter)/Diastolic aortic diameter] (SBP: Systolic blood pressure, DBP: Diastolic blood pressure).
- Aortic distensibility: 2 × (Systolic aortic diameter − Diastolic aortic diameter)/[(Diastolic aortic diameter) × (Aortic pulse pressure)].
- Aortic strain (%): [(Systolic aortic diameter − Diastolic aortic diameter) × 100]/Diastolic aortic diameter.

Aortic diameters were measured at the level of the ascending aorta approximately 3 cm above the aortic valve using M-mode echocardiography guided by two-dimensional imaging, according to standard echocardiographic recommendations. Measurements were obtained during systole and diastole from the parasternal long-axis view.

### 2.4. Statistical Analysis

The statistical analyses were performed using the Statistical Package for the Social Sciences (SPSS), version 25.0. In descriptive analyses, categorical variables were presented as numbers (*n*) and percentages (%), while continuous variables were expressed as mean ± standard deviation (SD) and median (interquartile range, IQR), as appropriate. The normality of distribution for continuous variables was assessed using the Kolmogorov–Smirnov test. For comparison between two independent groups, the Independent Samples *t*-test was used for normally distributed variables, whereas the Mann–Whitney U test was applied for non-normally distributed variables.

Relationships between continuous variables were evaluated using Spearman correlation analysis. Correlation strength was interpreted according to commonly accepted thresholds. To evaluate the effects of independent variables on dependent variables, linear regression analyses were conducted. A two-sided *p*-value < 0.05 was considered statistically significant. Cases with missing data on key outcome or predictor variables were excluded from the relevant analyses (listwise deletion). No imputation techniques were applied due to the low frequency of missing data and the retrospective nature of the study.

Linear regression analyses were performed using Suboxone use duration as the independent variable to explore its relationship with selected echocardiographic, hematological, and biochemical parameters.

Because multiple correlation analyses were performed, the results should be interpreted as exploratory and hypothesis-generating rather than confirmatory. No formal correction for multiple comparisons was applied due to the exploratory design of the study.

## 3. Results

A total of 280 subjects were included in the study, consisting of 130 intravenous (IV) drug users and 150 healthy controls. The gender distribution was similar between the IV drug user group and the control group (Male/Female: 129/1 vs. 147/3). The distribution of demographic, clinical, laboratory, and echocardiographic parameters among the groups is presented in Table 1. Compared to the control group, IV drug users had significantly higher levels of white blood cell count (WBC), neutrophils, uric acid, C-reactive protein (CRP), pulmonary artery pressure, inferior vena cava index (VCI), and aortic distensibility, while aortic stiffness was significantly lower (*p* < 0.001).

When grouped based on Suboxone use duration relative to 18 months, 45 patients had used Suboxone for ≤18 months, and 85 patients had used it for >18 months. The distribution of parameters according to Suboxone use duration is provided in Table 2. Patients with longer Suboxone use exhibited significantly higher diastolic blood pressure, mean arterial pressure, and pulmonary artery pressure (*p* = 0.044, *p* = 0.046, and *p* = 0.022, respectively).

The relationships between age, body mass index (BMI), Suboxone use duration, aortic distensibility, aortic stiffness, and other parameters in IV drug users are shown in Table 3. Age was found to have a strong correlation with systolic blood pressure, diastolic blood pressure, and mean arterial pressure (MAP); a weak correlation with LDL cholesterol, triglycerides, left atrial diameter (LA), aortic diameter, heart rate, QT interval, and iCEB; and a moderate correlation with aortic distensibility, aortic stiffness, and aortic strain (*p* < 0.05). BMI showed weak correlations with SBP, DBP, MAP, WBC, monocytes, hemoglobin, LDL, CRP, aortic distensibility, aortic stiffness, and aortic strain; and weak to moderate correlations with triglycerides and LA (*p* < 0.05). A weak negative correlation was observed between Suboxone use duration and ferritin levels (r = −0.231; *p* = 0.008). Aortic distensibility and aortic stiffness demonstrated moderate correlations with systolic blood pressure and left atrial diameter, and moderate-to-strong correlations with diastolic blood pressure, mean arterial pressure, and LDL cholesterol levels; weak correlations with hemoglobin and triglycerides; a strong correlation with aortic diameter; and a very strong correlation with aortic strain (*p* < 0.05).

In patients with long-term IV drug use, associations between age, BMI, aortic distensibility, aortic stiffness, and other parameters are presented in Table 4. Age showed a very strong correlation with SBP and MAP; a strong correlation with DBP; a moderate correlation with triglycerides; and weak to moderate correlations with left ventricular end-diastolic diameter (LVEDD), left ventricular end-systolic diameter (LVESD), aortic diameter, and iCEB; weak correlations with LA, heart rate, and QRS duration; and good correlations with aortic distensibility, aortic stiffness, and aortic strain (*p* < 0.05). BMI showed weak to moderate correlations with SBP, DBP, MAP, WBC, monocytes, uric acid, LDL, and triglycerides (*p* < 0.05). Aortic distensibility and aortic stiffness were moderately correlated with SBP, DBP, MAP, and LA, showed weaker associations with lipid parameters and left ventricular dimensions, and exhibited a strong correlation with aortic diameter and a very strong correlation with aortic strain (*p* < 0.05).

Linear regression analysis was performed to assess the effect of Suboxone use duration on echocardiographic, hematological, and biochemical parameters. Suboxone use duration did not show a statistically significant effect on echocardiographic parameters. However, a statistically significant effect was observed on red cell distribution width (Unstandardized B [95% CI]: 0.009 [0.000–0.018]; *p* = 0.042).

## 4. Discussion

Our study provides valuable insights into the cardiovascular implications of opioid use, particularly focusing on aortic stiffness and distensibility among individuals undergoing buprenorphine/naloxone maintenance therapy. These results suggest that chronic opioid exposure, despite its known cardiovascular risks, may be associated with altered aortic elasticity. Additionally, no significant association was found between aortic stiffness measures and the index of cardiac electrophysiological balance. In our correlation analyses, aortic elasticity indices demonstrated very strong associations with aortic strain. Because aortic strain and distensibility are derived from similar diameter-based echocardiographic calculations, this strong correlation was expected. These values reflect the formula-based interdependence of these parameters rather than independent physiological phenomena.

The duration of Suboxone use presented notable associations with specific cardiovascular parameters. Patients with longer Suboxone use exhibited significantly higher diastolic blood pressure, mean arterial pressure, and pulmonary artery pressure. These observations suggest that prolonged exposure to buprenorphine/naloxone may influence hemodynamic regulation, potentially contributing to elevated cardiovascular risk over time. Intravenous opioid users exhibited higher aortic distensibility and lower aortic stiffness compared with controls, which contrasts with the well-established association between opioid use and increased cardiovascular risk. Because the control group was age-matched to the opioid group, these differences cannot be explained solely by age. Instead, they may reflect physiological effects related to opioid exposure or maintenance therapy. Buprenorphine/naloxone may influence autonomic regulation by reducing sympathetic activity and promoting vasodilatory responses through endothelial nitric oxide-mediated pathways. Such mechanisms could potentially result in transient increases in arterial compliance in relatively young and clinically stable individuals receiving maintenance therapy. Alternatively, early adaptive vascular remodeling in response to chronic systemic stress may initially manifest as increased distensibility before progressing to arterial stiffening in later stages. Chronic opioid use, particularly buprenorphine/naloxone, may exert autonomic effects that promote vasodilation, reduce sympathetic tone, or influence endothelial nitric oxide activity, leading to transient increases in arterial compliance. Alternatively, compensatory remodeling in response to systemic inflammation or repeated hemodynamic stress could initially present as increased distensibility before progressing to stiffness in later disease stages. Lastly, measurement variability or methodological constraints inherent to retrospective data may have contributed to these findings.

In our study, no significant differences were observed in iCEB and iCEBc ratios between opioid users and healthy controls. However, a significant positive correlation was found between the duration of Suboxone use and both iCEB and iCEBc ratios. This finding suggests that prolonged exposure to Suboxone may lead to more pronounced changes in cardiac repolarization processes. Literature suggests that iCEB ratio could potentially serve as a marker for ventricular arrhythmias and sudden cardiac death. Liu et al. reported that an increase in iCEB and iCEBc ratios is associated with myocardial electrical instability, especially in conditions such as substance use disorders, where these ratios may have prognostic significance [15]. Our study supports this hypothesis, showing a trend toward increased iCEB and iCEBc ratios with longer Suboxone usage, suggesting a potential association between longer therapy duration and electrophysiological parameters, although the clinical significance of this finding remains uncertain. In this context, iCEB and iCEBc ratios should be considered as non-invasive, easily accessible monitoring parameters for assessing cardiac electrical stability in opioid-dependent individuals [16]. Regular monitoring of these parameters, particularly in those on long-term Suboxone therapy, may help predict potential arrhythmic events. Also, subgroup analyses revealed a moderate positive correlation between age and iCEB, particularly among patients with longer Suboxone use. Accordingly, these findings should be considered hypothesis-generating rather than conclusive, highlighting the need for prospective studies to clarify the clinical significance of iCEB and iCEBc alterations in opioid-dependent populations.

The lack of a significant association between aortic stiffness and iCEB/iCEBc may reflect the inherent complexity of arrhythmogenesis, which is influenced by multiple interacting factors, including myocardial substrate, autonomic tone, electrolyte balance, and systemic inflammation—many of which may not be directly captured by arterial stiffness alone. Additionally, although iCEB has shown promise as a non-invasive arrhythmic risk marker, its sensitivity in detecting subtle electrophysiological changes associated with vascular remodeling remains to be fully validated. This may be particularly true in relatively young and clinically stable populations, such as our cohort.

Our analysis revealed a significant negative correlation between aortic stiffness and LDL cholesterol levels. This inverse relationship aligns with certain studies indicating complex interactions between lipid profiles and arterial elasticity [17]. While elevated LDL cholesterol is traditionally viewed as a risk factor for atherosclerosis and increased arterial stiffness, some populations exhibit paradoxical associations, underscoring the multifaceted nature of lipid metabolism’s impact on vascular health. It should be noted, however, that our subgroup comparisons based on Suboxone use duration provide only a cross-sectional snapshot rather than true longitudinal data. The observed associations may be influenced by unmeasured baseline differences rather than changes over time.

Age emerged as a critical determinant of vascular properties in our cohort. We noted a moderate correlation between advancing age and increased aortic stiffness, accompanied by decreased aortic strain. These findings are consistent with established literature indicating that aging is associated with structural and functional changes in the arterial wall, leading to reduced elasticity and heightened stiffness. Such age-related vascular remodeling underscores the importance of considering patient age when evaluating cardiovascular risk profiles.

Our findings align with existing literature highlighting the cardiovascular risks associated with opioid use. Studies have reported increased rates of cardiovascular disease among patients prescribed opioids, emphasizing the need for comprehensive cardiovascular risk assessment in this population [18,19]. Additionally, research has demonstrated that aortic stiffness is a significant predictor of cardiovascular events, further underscoring the importance of monitoring vascular health in opioid users.

Collectively, our findings suggest that prolonged buprenorphine/naloxone maintenance therapy may be associated with measurable alterations in vascular elasticity and cardiac electrophysiological balance. These changes, although exploratory in nature, may reflect early vascular remodeling and compensatory adaptation in opioid-dependent individuals.

### Limitations

This study has several limitations. First, the retrospective nature of the study introduces the potential for selection bias and limits our ability to control for unmeasured confounders. As such, causal inferences cannot be definitively established, and the associations observed should be interpreted as exploratory. Second, although statistically significant differences were observed in major outcomes such as aortic stiffness and distensibility, the relatively small sample size and retrospective design may have limited the detection of more subtle differences in secondary outcomes, including iCEB and iCEBc. The potential influence of unmeasured confounders such as lifestyle factors, comorbidities, and concurrent medications further limits causal inference. Additionally, we acknowledge the lack of multivariate regression analysis as a limitation. Although our study identified several statistically significant associations through correlation and univariate regression methods, we were unable to conduct full multivariate modeling due to the limited sample size and the exploratory nature of the design. Moreover, the study sample included only individuals receiving stable buprenorphine/naloxone maintenance therapy. These patients may differ from the broader OUD population in terms of clinical stability, adherence to medical follow-up, and access to care. As such, the findings may not be generalizable to opioid users who are not in treatment or who present with higher degrees of social and medical vulnerability.

Because of the relatively limited sample size and the exploratory nature of the study, full multivariate modeling was not performed. Therefore, the associations reported in this study represent unadjusted relationships and should be interpreted as hypothesis-generating rather than independent causal associations.

Another limitation of the present study is the lack of detailed information regarding the exact dosage of buprenorphine/naloxone therapy. Because the study was retrospective and dosage adjustments varied during follow-up, consistent dosage data were not available for all participants. Therefore, the potential influence of medication dosage on vascular and electrophysiological parameters could not be evaluated. Additionally, the exclusion of active smokers resulted in a selected subgroup of opioid-dependent individuals who may represent a more clinically stable population under regular medical follow-up. This selection may limit the generalizability of the findings to the broader population of individuals with opioid use disorder.

Because the control group was intentionally selected to exclude smoking, alcohol consumption, and other psychoactive substance use in order to minimize confounding factors affecting vascular elasticity, the control population may represent a healthier subset of the general population. This selection may have exaggerated differences between groups and should be considered when interpreting the results.

The present study did not include an a priori sample size calculation because of its retrospective design. However, considering the primary outcome variable (aortic stiffness), the observed between-group difference was substantial and statistically significant (*p* < 0.001). Given the sample sizes of the opioid user group (*n* = 130) and the control group (*n* = 150), the study likely had adequate statistical power to detect clinically meaningful differences in aortic elasticity parameters. Nevertheless, future prospective studies with predefined sample size calculations are required to confirm these findings and to evaluate additional cardiovascular parameters with greater precision.

Despite these limitations, we believe our results provide meaningful insights and generate hypotheses for future prospective studies with larger, multicenter cohorts. Finally, long-term follow-up data were not available, limiting the assessment of clinical cardiovascular outcomes.

## 5. Conclusions

In conclusion, opioid-dependent individuals receiving buprenorphine/naloxone maintenance therapy exhibit alterations in aortic elasticity and cardiac electrophysiological balance. These findings underscore the importance of incorporating non-invasive vascular and electrocardiographic assessment into cardiovascular surveillance strategies for patients receiving long-term opioid maintenance therapy. Future prospective, multicenter studies are warranted to validate these observations and to clarify their long-term clinical implications.

## Figures and Tables

**Table 1:** Distribution of data in IV drug users and control groups.

	IV Drug Users (*n* = 130)	Control (*n* = 150)	Test Value	*p* Value
Age	28.00 (25.00–31.00)	28.00 (25.00–31.00)	0.138 *	0.890
Systolic Blood Pressure	110.00 (106.00–115.00)	109.00 (106.00–115.00)	0.578 *	0.563
Diastolic Blood Pressure	70.00 (65.75–74.00)	69.00 (65.00–74.25)	0.593 *	0.553
Mean arterial pressure	83.00 (79.00–87.75)	82.66 (78.33–88.08)	0.633 *	0.527
White blood cell count	7.58 (6.56–8.89)	6.86 (6.16–7.56)	4.540 *	<0.001
Platelet	244.00 (197.75–303.75)	243.00 (198.00–309.00)	0.317 *	0.751
Lymphocyte	2.31 (1.90–2.94)	2.42 (1.97–2.72)	0.096 *	0.923
Monocyte	0.51 (0.39–0.67)	0.51 (0.38–0.65)	0.238 *	0.812
Neutrophil	4.36 (3.52–5.21)	3.79 (3.29–4.18)	4.895 *	<0.001
Hemoglobin	14.43 ± 1.33	14.52 ± 1.15	0.578 **	0.564
RDW	13.90 (13.20–14.62)	13.80 (13.20–14.60)	0.136 *	0.892
Albumin	43.50 (41.27–45.80)	43.95 (42.20–45.80)	1.846 *	0.065
Uric acid	5.20 (4.50–6.12)	4.80 (4.30–5.20)	4.506 *	<0.001
LDL	86.50 (63.00–101.25)	86.50 (64.50–101.00)	0.138 *	0.891
HDL	43.00 (37.75–48.25)	43.00 (38.00–49.00)	0.055 *	0.956
Triglyceride	110.50 (70.75–163.50)	109.50 (69.75–132.00)	1.466 *	0.143
Ferritin	44.00 (27.00–59.25)	43.00 (26.75–56.00)	1.005 *	0.315
CRP	6.00 (3.00–10.25)	3.00 (3.00–4.00)	6.735 *	<0.001
TSH	2.05 (1.25–2.89)	2.20 (1.73–2.86)	1.797 *	0.072
LVEDD	44.00 (44.00–46.00)	44.00 (43.75–46.00)	0.610 *	0.542
LVESD	27.00 (27.00–28.00)	27.00 (27.00–28.00)	0.331 *	0.741
LA	28.00 (28.00–30.00)	28.00 (28.00–30.00)	0.420 *	0.675
Aort Diameter	24.00 (24.00–27.00)	24.00 (23.75–27.00)	0.380 *	0.704
Pulmonary Artery Pressure	23.00 (19.00–26.00)	15.00 (13.00–18.00)	10.758 *	<0.001
TAPSE	24.00 (23.00–25.00)	24.00 (23.00–25.00)	0.659 *	0.510
VCI (expirium) Diameter	1.58 ± 0.09	1.53 ± 0.09	4.291 **	<0.001
Aortic distensibility	0.019 (0.015–0.023)	0.015 (0.012–0.018)	6.103 *	<0.001
Aortic stiffness	52.31 (42.97–63.65)	64.66 (53.18–78.98)	6.103 *	<0.001
Septal E/E’ Ratio	0.73 (0.65–0.81)	0.71 (0.65–0.77)	1.221 *	0.222
Lateral E/E’ ratio	1.28 (1.22–1.40)	1.32 (1.24–1.40)	1.804 *	0.071
PR interval (sec)	0.14 (0.13–0.15)	0.14 (0.13–0.15)	0.107 *	0.915
QRS duration (sec)	0.09 (0.08–0.09)	0.09 (0.08–0.09)	0.436 *	0.663
QTc duration (sec)	0.38 (0.38–0.39)	0.38 (0.38–0.39)	0.250 *	0.802
iCEB	3.83 (3.61–4.12)	3.83 (3.65–4.14)	0.324 *	0.746
iCEBc	4.25 (3.98–4.54)	4.25 (4.00–4.55)	0.371 *	0.710

Median (IQR); Mean ± SD. *: Mann–Whitney U Test. **: Independent samples *t* test. Abbreviations: LVEDD = Left ventricular end-diastolic diameter; LVESD = Left ventricular end-systolic diameter; TAPSE = Tricuspid annular plane systolic excursion; VCI = Vena cava inferior; RDW: Red cell distribution width; CRP: C-Reactive protein; TSH: Thyroid Stimulating Hormone; iCEB: Index of Cardiac Electrophysiological Balance; iCEBc: Corrected Index of Cardiac Electrophysiological Balance; LA: Left atrium; HDL: High density lipoprotein; LDL: Low density lipoprotein.

**Table 2:** Distribution of data according to duration of suboxone use.

	IV Suboxone ≤ 18 Months (*n* = 45)	IV Suboxone > 18 Months (*n* = 85)	Test Value	*p* Value
Age	28.00 (25.00–30.00)	29.00 (25.00–32.00)	0.714 *	0.475
Systolic Blood Pressure	108.00 (105.0–113.00)	111.00 (106.50–115.50)	1.880 *	0.060
Diastolic Blood Pressure	67.00 (65.00–72.00)	70.00 (66.00–75.00)	2.015 *	0.044
Mean arterial pressure	80.66 (78.66–85.83)	83.66 (79.66–88.33)	1.997 *	0.046
White blood cell count	7.43 (6.14–8.01)	7.63 (6.64–9.20)	1.353 *	0.176
Platelet	235.00 (189.00–283.00)	248.00 (208.00–314.00)	1.483 *	0.138
Lymphocyte	2.17 (1.81–2.79)	2.42 (1.96–2.99)	1.476 *	0.140
Monocyte	0.50 (0.42–0.63)	0.51 (0.38–0.69)	0.027 *	0.979
Neutrophil	4.05 (3.40–4.94)	4.52 (3.56–5.46)	1.385 *	0.166
Hemoglobin	14.44 ± 1.43	14.42 ± 1.28	0.084 **	0.934
RDW	13.60 (12.95–14.30)	14.10 (13.20–14.75)	1.484 *	0.138
Albumin	43.20 (41.00–45.80)	43.70 (41.35–45.75)	0.296 *	0.767
Uric acid	4.90 (4.30–5.80)	5.30 (4.75–6.25)	1.697 *	0.090
LDL	86.00 (63.00–104.50)	87.00 (64.00–99.00)	0.303 *	0.762
HDL	42.00 (37.50–49.00)	43.00 (37.50–48.00)	0.338 *	0.735
Triglyceride	111.00 (70.50–164.00)	110.00 (70.50–164.00)	0.267 *	0.790
Ferritin	46.00 (34.50–76.50)	39.00 (25.50–57.50)	1.606 *	0.108
CRP	7.00 (3.00–12.85)	6.00 (3.00–10.00)	0.623 *	0.534
TSH	2.05 (1.27–2.89)	2.05 (1.21–2.83)	0.210 *	0.833
LVEDD	44.00 (44.00–46.50)	44.00 (44.00–46.00)	1.024 *	0.306
LVESD	28.00 (27.00–30.00)	27.00 (27.00–28.00)	1.441 *	0.149
LA	29.00 (28.00–30.00)	28.00 (28.00–30.00)	1.005 *	0.315
Aort Diameter	24.00 (24.00–26.50)	24.00 (23.00–27.00)	0.251 *	0.802
Pulmonary Artery Pressure	22.00 (17.50–26.00)	23.00 (19.00–27.00)	2.289 *	0.022
TAPSE	24.00 (23.00–25.00)	24.00 (23.50–25.00)	0.225 *	0.822
VCI (expirium) Diameter	1.57 ± 0.09	1.58 ± 0.09	0.836 **	0.405
Aortic distensibility	0.018 (0.015–0.021)	0.019 (0.016–0.023)	0.690 *	0.490
Aortic stiffness	53.04 (45.73–65.69)	52.04 (42.27–62.49)	0.690 *	0.490
Septal E/E’ Ratio	0.74 (0.66–0.84)	0.73 (0.64–0.79)	1.045 *	0.296
Lateral E/E’ ratio	1.27 (1.21–1.33)	1.29 (1.23–1.41)	1.380 *	0.168
PR interval (sec)	0.14 (0.13–0.16)	0.14 (0.13–0.15)	1.702 *	0.089
QRS duration (sec)	0.09 (0.08–0.09)	0.09 (0.08–0.09)	0.324 *	0.746
QTc duration (sec)	0.39 (0.38–0.39)	0.38 (0.38–0.39)	1.065 *	0.287
iCEB	3.80 (3.55–4.09)	3.88 (3.62–4.14)	0.690 *	0.490
iCEBc	4.21 (3.98–4.52)	4.25 (3.98–4.56)	0.208 *	0.835

Median (IQR); Mean ± SD. *: Mann–Whitney U Test. **: Independent samples *t* test. Abbreviations: LVEDD = Left ventricular end-diastolic diameter; LVESD = Left ventricular end-systolic diameter; TAPSE = Tricuspid annular plane systolic excursion; VCI = Vena cava inferior; RDW: Red cell distribution width; CRP: C-Reactive protein; TSH: Thyroid Stimulating Hormone; iCEB: Index of Cardiac Electrophysiological Balance; iCEBc: Corrected Index of Cardiac Electrophysiological Balance; LA: Left atrium; HDL: High density lipoprotein; LDL: Low density lipoprotein.

**Table 3:** Correlation of data with age, BMI, suboxone use, aortic distensibility and aortic stiffness in groups.

		IV Drug Users				
		Age	BMI	Suboxone Usage Duration	Aortic Distensibility	Aortic Stiffness
Systolic Blood Pressure	r *p*	0.821 <0.001	0.251 0.004	0.131 0.130	−0.449 <0.001	0.449 <0.001
Diastolic Blood Pressure	r *p*	0.776 <0.001	0.277 0.001	0.147 0.095	−0.367 <0.001	0.367 <0.001
Mean arterial pressure	r *p*	0.800 <0.001	0.275 0.002	0.144 0.101	−0.397 <0.001	0.397 <0.001
White blood cell count	r *p*	0.049 0.580	0.209 0.017	0.066 0.455	−0.098 0.266	0.098 0.266
Platelet	r *p*	−0.028 0.751	0.098 0.267	0.122 0.168	0.025 0.775	−0.025 0.775
Lymphocyte	r *p*	0.044 0.617	0.097 0.272	0.128 0.145	−0.045 0.614	0.045 0.614
Monocyte	r *p*	0.056 0.526	0.217 0.013	−0.059 0.503	−0.017 0.852	0.017 0.852
Neutrophil	r *p*	0.023 0.793	0.143 0.105	0.061 0.492	−0.074 0.406	0.074 0.406
Hemoglobin	r *p*	0.071 0.422	0.196 0.026	−0.011 0.901	−0.177 0.044	0.177 0.044
Albumin	r *p*	−0.143 0.106	0.049 0.577	0.069 0.438	0.085 0.339	−0.085 0.339
Uric acid	r *p*	−0.015 0.864	0.183 0.037	0.057 0.522	−0.053 0.552	0.053 0.552
LDL	r *p*	0.243 0.005	0.278 0.001	−0.010 0.909	−0.307 <0.001	0.307 <0.001
HDL	r *p*	0.085 0.338	0.023 0.797	0.016 0.859	−0.067 0.448	0.067 0.448
Triglyceride	r *p*	0.277 0.001	0.304 <0.001	0.001 0.999	−0.202 0.021	0.202 0.021
Ferritin	r *p*	−0.066 0.453	−0.113 0.202	−0.231 0.008	0.058 0.513	−0.058 0.513
CRP	r *p*	−0.019 0.830	−0.230 0.009	−0.159 0.070	0.160 0.068	−0.160 0.068
TSH	r *p*	−0.091 0.301	0.062 0.482	−0.086 0.332	0.066 0.457	−0.066 0.457
LVEDD	r *p*	0.129 0.143	0.132 0.135	−0.065 0.466	−0.127 0.151	0.127 0.151
LVESD	r *p*	0.136 0.124	0.123 0.162	−0.093 0.294	−0.158 0.072	0.158 0.072
LA	r *p*	0.276 0.002	0.310 <0.001	−0.073 0.407	−0.488 <0.001	0.488 <0.001
Aort Diameter	r *p*	0.200 0.023	0.140 0.112	−0.025 0.782	−0.650 <0.001	0.650 <0.001
Pulmonary Artery Pressure	r *p*	0.072 0.414	0.017 0.849	0.084 0.343	−0.066 0.456	0.066 0.456
TAPSE	r *p*	−0.153 0.082	−0.103 0.245	0.042 0.634	0.066 0.458	0.066 0.458
Aortic distensibility	r *p*	−0.555 <0.001	−0.215 0.015	0.074 0.402		
Aortic stiffness	r *p*	0.555 <0.001	0.215 0.015	−0.074 0.402		
Septal E/E’ Ratio	r *p*	0.004 0.966	0.111 0.208	−0.067 0.451	−0.166 0.060	0.166 0.060
Lateral E/E’ ratio	r *p*	−0.129 0.142	−0.166 0.058	0.092 0.296	0.078 0.377	−0.078 0.377
Heart rate	r *p*	−0.203 0.021	0.083 0.348	−0.109 0.219	0.068 0.441	−0.068 0.441
PR interval (sec)	r *p*	0.052 0.555	0.157 0.075	−0.078 0.378	−0.065 0.462	0.065 0.462
QRS duration (sec)	r *p*	−0.094 0.285	−0.018 0.837	0.081 0.362	0.046 0.604	−0.046 0.604
QT interval (sec)	r *p*	0.175 0.046	−0.014 0.878	0.080 0.367	−0.074 0.404	0.074 0.404
QTc duration (sec)	r *p*	0.072 0.146	0.123 0.163	−0.040 0.655	−0.086 0.329	0.086 0.329
iCEB	r *p*	0.214 0.015	0.023 0.792	−0.030 0.735	−0.086 0.329	0.086 0.329
iCEBc	r *p*	0.086 0.333	0.068 0.442	−0.082 0.351	−0.053 0.550	0.053 0.550
Aortic Strain	r *p*	−0.553 <0.001	−0.232 0.008	0.068 0.440	0.982 <0.001	−0.982 <0.001

r = Spearman’s correlation coefficient. Abbreviations: LVEDD = Left ventricular end-diastolic diameter; LVESD = Left ventricular end-systolic diameter; TAPSE = Tricuspid annular plane systolic excursion; CRP: C-Reactive protein; TSH: Thyroid Stimulating Hormone; iCEB: Index of Cardiac Electrophysiological Balance; iCEBc: Corrected Index of Cardiac Electrophysiological Balance; LA: Left atrium; HDL: High density lipoprotein; LDL: Low density lipoprotein.

**Table 4:** Correlation of data with age, BMI, aortic distensibility, and aortic stiffness in suboxone duration groups.

		IV Suboxone > 18 Months			
		Age	BMI	Aortic Distensibility	Aortic Stiffness
Systolic Blood Pressure	r *p*	0.778 <0.001	0.332 0.002	−0.559 <0.001	0.559 <0.001
Diastolic Blood Pressure	r *p*	0.732 <0.001	0.390 <0.001	−0.499 <0.001	0.499 <0.001
Mean arterial pressure	r *p*	0.754 <0.001	0.385 <0.001	−0.525 <0.001	0.525 <0.001
White blood cell count	r *p*	0.011 0.921	0.280 0.010	−0.150 0.171	0.150 0.171
Platelet	r *p*	−0.054 0.625	0.089 0.416	0.049 0.656	−0.049 0.656
Lymphocyte	r *p*	0.098 0.373	0.147 0.178	−0.102 0.352	0.102 0.352
Monocyte	r *p*	0.085 0.438	0.295 0.006	−0.180 0.100	0.180 0.100
Neutrophil	r *p*	−0.035 0.752	0.204 0.061	−0.120 0.275	0.120 0.275
Hemoglobin	r *p*	0.155 0.156	0.179 0.100	−0.205 0.060	0.205 0.060
Albumin	r *p*	−0.179 0.101	0.025 0.820	0.158 0.148	−0.158 0.148
Uric acid	r *p*	−0.018 0.867	0.229 0.035	−0.055 0.616	0.055 0.616
LDL	r *p*	0.331 0.002	0.217 0.046	−0.316 0.003	0.316 0.003
HDL	r *p*	0.165 0.130	0.038 0.730	−0.117 0.286	0.117 0.286
Triglyceride	r *p*	0.425 <0.001	0.231 0.033	−0.299 0.005	0.299 0.005
Ferritin	r *p*	−0.105 0.340	−0.154 0.160	−0.011 0.923	0.011 0.923
CRP	r *p*	−0.056 0.608	−0.166 0.129	0.044 0.686	−0.044 0.686
TSH	r *p*	−0.127 0.248	0.107 0.332	0.046 0.675	−0.046 0.675
LVEDD	r *p*	0.324 0.002	0.191 0.080	−0.233 0.032	0.233 0.032
LVESD	r *p*	0.326 0.002	0.200 0.067	−0.256 0.018	0.256 0.018
LA	r *p*	0.294 0.006	0.301 0.005	−0.473 <0.001	0.473 <0.001
Aort Diameter	r *p*	0.323 0.003	0.120 0.272	−0.675 <0.001	0.675 <0.001
Pulmonary Artery Pressure	r *p*	0.176 0.106	0.009 0.932	−0.178 0.104	0.178 0.104
TAPSE	r *p*	−0.128 0.244	−0.008 0.944	−0.018 0.868	0.018 0.868
Aortic distensibility	r *p*	−0.669 <0.001	−0.138 0.209		
Aortic stiffness	r *p*	0.669 <0.001	0.138 0.209		
Septal E/E’ Ratio	r *p*	0.036 0.743	0.040 0.716	−0.128 0.243	0.128 0.243
Lateral E/E’ ratio	r *p*	−0.120 0.275	−0.150 0.170	0.142 0.195	−0.142 0.195
Heart rate	r *p*	−0.254 0.019	0.112 0.308	0.161 0.141	−0.161 0.141
PR interval (sec)	r *p*	−0.005 0.967	0.111 0.311	0.027 0.808	−0.027 0.808
QRS duration (sec)	r *p*	−0.237 0.029	−0.005 0.967	0.136 0.213	−0.136 0.213
QT interval (sec)	r *p*	0.193 0.076	−0.123 0.260	−0.095 0.386	0.095 0.386
QTc duration (sec)	r *p*	0.057 0.607	−0.059 0.593	−0.026 0.813	0.026 0.813
iCEB	r *p*	0.367 0.001	−0.072 0.510	−0.188 0.084	0.188 0.084
iCEBc	r *p*	0.211 0.053	0.002 0.988	−0.129 0.238	0.129 0.238
Aortic Strain	r *p*	−0.648 <0.001	−0.156 0.153	0.984 <0.001	−0.984 <0.001

r = Spearman’s correlation coefficient. Abbreviations: LVEDD = Left ventricular end-diastolic diameter; LVESD = Left ventricular end-systolic diameter; TAPSE = Tricuspid annular plane systolic excursion; CRP: C-Reactive protein; TSH: Thyroid Stimulating Hormone; iCEB: Index of Cardiac Electrophysiological Balance; iCEBc: Corrected Index of Cardiac Electrophysiological Balance; LA: Left atrium; HDL: High density lipoprotein; LDL: Low density lipoprotein.

## Data Availability

The data presented in this study are available on request from the corresponding author.

## Abbreviations

ABPM	Ambulatory blood pressure monitoring
BMI	Body mass index
CRP	C-reactive protein
DBP	Diastolic blood pressure
ECG	Electrocardiography
HR	Heart rate
iCEB	Index of cardiac electrophysiological balance (QT/QRS)
iCEBc	Corrected index of cardiac electrophysiological balance (QTc/QRS)
IV	Intravenous
LA	Left atrium
LDL	Low-density lipoprotein
LVEDD	Left ventricular end-diastolic diameter
LVESD	Left ventricular end-systolic diameter
MAP	Mean arterial pressure
OUD	Opioid use disorder
SBP	Systolic blood pressure
TTE	Transthoracic echocardiography
WBC	White blood cell count

## References

[1] Mokdad A.H., Ballestros K., Echko M., Glenn S., Olsen H.E., Mullany E., Lee A., Khan A.R., Ahmadi A., Ferrari A.J. (2018). The State of US Health, 1990–2016: Burden of Diseases, Injuries, and Risk Factors Among US States. JAMA.

[2] Friedman J., Akre S. (2021). COVID-19 and the drug overdose crisis: Uncovering the deadliest months in the United States, January–July 2020. Am. J. Public Health.

[3] Florence C.S., Zhou C., Luo F., Xu L. (2016). The Economic Burden of Prescription Opioid Overdose, Abuse, and Dependence in the United States, 2013. Med. Care.

[4] Montiel Ishino F.A., McNab P.R., Gilreath T., Salmeron B., Williams F. (2020). A comprehensive multivariate model of biopsychosocial factors associated with opioid misuse and use disorder in a 2017–2018 United States national survey. BMC Public Health.

[5] Alexander G.C., Stoller K.B., Haffajee R.L., Saloner B. (2020). An epidemic in the midst of a pandemic: Opioid use disorder and COVID-19. Ann. Intern. Med..

[6] Mondal A., Dadana S., Parmar P., Mylavarapu M., Dong Q., Butt S.R., Kali A., Bollu B., Desai R. (2024). Association of Cannabis Use Disorder with Major Adverse Cardiac and Cerebrovascular Events in Older Non-Tobacco Users: A Population-Based Analysis. Med. Sci..

[7] Nosyk B., Min J.E., Homayra F., Kurz M., Guerra-Alejos B.C., Yan R., Piske M., Seaman S.R., Bach P., Greenland S. (2024). Buprenorphine/naloxone vs methadone for the treatment of opioid use disorder. JAMA.

[8] Vallée A. (2023). Association between tobacco smoking and alcohol consumption with arterial stiffness. J. Clin. Hypertens..

[9] Gan W.Q., Buxton J.A., Scheuermeyer F.X., Palis H., Zhao B., Desai R., Janjua N.Z., Slaunwhite A.K. (2021). Risk of cardiovascular diseases in relation to substance use disorders. Drug Alcohol Depend..

[10] Moulakakis K.G., Pitros C.F., Theodosopoulos I.T., Mylonas S.N., Kakisis J.D., Manopoulos C., Kadoglou N.P.E. (2024). Arterial Stiffness and Aortic Aneurysmal Disease—A Narrative Review. Vasc. Health Risk Manag..

[11] Szabó P.L., Dostal C., Pilz P.M., Hamza O., Acar E., Watzinger S., Mathew S., Kager G., Hallström S., Podesser B.K. (2021). Remote Ischemic Perconditioning Ameliorates Myocardial Ischemia and Reperfusion-Induced Coronary Endothelial Dysfunction and Aortic Stiffness in Rats. J. Cardiovasc. Pharmacol. Ther..

[12] Szołtysek-Bołdys I., Zielińska-Danch W., Sarecka-Hujar B., Słodczyk-Mańkowska E., Kozar-Konieczna A., Sobczak A. (2023). Does Alcohol Withdrawal Influence Arterial Stiffness and Classical Risk Factors for Cardiovascular Disease for Persons With Alcohol Use Disorder?. Alcohol Alcohol..

[13] Alsancak Y., Sahın A.T., Gurbuz A.S., Sertdemir A.L., Icli A., Akilli H., Duzenli M.A. (2020). Index of cardiac-electrophysiological balance and the effects of thrombolytic therapy on the electrocardiogram of patients with pulmonary embolism. Rev. Assoc. Méd. Bras..

[14] Mozos I. (2015). The link between ventricular repolarization variables and arterial function. J. Electrocardiol..

[15] Liu Q., Yuan X., Sheng C., Cai W., Geng X., Liu H., Song S. (2024). Effect of long-term use of antipsychotics on the ventricular repolarization index. BMC Psychiatry.

[16] Gonçalves M.A., Pedro J.M., Silva C., Magalhães P., Brito M. (2024). Influence of cigarette smoking on the Index of Cardiac Electrophysiological Balance in apparently healthy Angolans. Glob. Cardiol. Sci. Pract..

[17] Baba M., Maris M., Jianu D., Luca C.T., Stoian D., Mozos I. (2023). The impact of the blood lipids levels on arterial stiffness. J. Cardiovasc. Dev. Dis..

[18] Chow S.L., Sasson C., Benjamin I.J., Califf R.M., Compton W.M., Oliva E.M., Robson C., Sanchez E.J. (2021). Opioid Use and Its Relationship to Cardiovascular Disease and Brain Health: A Presidential Advisory From the American Heart Association. Circulation.

[19] Krantz M.J., Palmer R.B., Haigney M.C.P. (2021). Cardiovascular Complications of Opioid Use: JACC State-of-the-Art Review. J. Am. Coll. Cardiol..

